# CircRNA Expression Pattern and ceRNA and miRNA–mRNA Networks Involved in Anther Development in the CMS Line of *Brassica campestris*

**DOI:** 10.3390/ijms20194808

**Published:** 2019-09-27

**Authors:** Yuwei Liang, Yuzhi Zhang, Liai Xu, Dong Zhou, Zongmin Jin, Huiyan Zhou, Sue Lin, Jiashu Cao, Li Huang

**Affiliations:** 1 Laboratory of Cell & Molecular Biology, Institute of Vegetable Science, Zhejiang University, Hangzhou 310058, China; 21716058@zju.edu.cn (Y.L.); 13914738691@163.com (Y.Z.); 11416052@zju.edu.cn (L.X.); 15574994586@163.com (D.Z.); 21816052@zju.edu.cn (Z.J.); ruby1zhou@126.com (H.Z.); jshcao@zju.edu.cn (J.C.); 2 Key Laboratory of Horticultural Plant Growth, Development and Quality Improvement, Ministry of Agriculture/Zhejiang Provincial Key Laboratory of Horticultural Plant Integrative Biology, Hangzhou 310058, China; 3 Institute of Life Sciences, Wenzhou University, Wenzhou 325000, China; iamkari@163.com

**Keywords:** cytoplasmic male sterility, CMS, Polima, *Brassica campestris*, whole-transcriptome sequencing, ceRNA, circRNA, miRNA, anther

## Abstract

Male-sterile plants provide an important breeding tool for the heterosis of hybrid crops, such as Brassicaceae. In the last decade, circular RNAs (circRNAs), as a novel class of covalently closed and single-stranded endogenous non-coding RNAs (ncRNAs), have received much attention because of their functions as “microRNA (miRNA) sponges” and “competing endogenous RNAs” (ceRNAs). However, the information about circRNAs in the regulation of male-sterility and anther development is limited. In this study, we established the Polima cytoplasm male sterility (CMS) line “Bcpol97-05A”, and the fertile line, “Bcajh97-01B”, in *Brassica* *campestris* L. ssp. *chinensis* Makino, syn. *B. rapa* ssp. *chinensis*, and performed RNA expression profiling comparisons between the flower buds of the sterile line and fertile line by whole-transcriptome sequencing. A total of 31 differentially expressed (DE) circRNAs, 47 DE miRNAs, and 4779 DE mRNAs were identified. By using Cytoscape, the miRNA-mediated regulatory network and ceRNA network were constructed, and the circRNA A02:23507399|23531438 was hypothesized to be an important circRNA regulating anther development at the post-transcriptional level. The gene ontology (GO) analysis demonstrated that miRNAs and circRNAs could regulate the orderly secretion and deposition of cellulose, sporopollenin, pectin, and tryphine; the timely degradation of lipids; and the programmed cell death (PCD) of tapetum cells, which play key roles in anther development. Our study revealed a new circRNA–miRNA–mRNA network, which is involved in the anther development of *B. campestris,* which enriched the understanding of CMS in flowering plants, and laid a foundation for further study on the functions of circRNAs and miRNAs during anther development.

## 1. Introduction

The male sterility of plants, including genic male sterility (GMS) and cytoplasmic male sterility (CMS), refers to their incapacity to generate normal functional pollen grains. CMS is an essential plant reproductive characteristic that can be used as a useful tool to exploit crop heterosis [1]. The utilization of heterosis has produced tremendous economic benefits in crop production worldwide, which means that the contribution of hybrid breeding to the world’s food supply is enormous [2]. CMS plants not only provide crucial breeding tools for the utilization of heterosis in hybrid crops, but also important materials to study the mechanisms underlying anther development. The genes that cause CMS are located in the mitochondrial genome and their expressions are controlled by nuclear genes. Polima (*pol*) CMS reported in radish is the first CMS system widely used for hybrid seed production. Studies on the molecular mechanisms of *Brassica napus* revealed that the chimeric mitochondrial gene *orf224* located upstream of and co-transcribed with *atp6*, is the causal gene of *pol* CMS [3,4], however, the molecular mechanisms of anther and pollen abortion caused by these mitochondrial genes are still unclear. In recent years, genome-wide differential RNA expression analysis has made it possible to study the transcriptome differences between the sterile and fertile lines in *pol* CMS [5,6], facilitating researchers to reveal the changes in the whole-transcriptome level during the biogenesis of CMS, and to promote the study of the molecular mechanisms and male reproductive development of flowering plants. For example, a study in *B. napus* has shown that the genes regulating pollen development by nuclear-mitochondrial interaction might be inhibited by energy deficits caused by *orf224*/*atp6* [5]. Meanwhile, increasing evidence has demonstrated that microRNAs (miRNAs) could act as important regulators of CMS systems in plants [7,8,9,10]. For example, some miRNAs and their targets, which may regulate flower bud development such as gma-miR156b/*GmSPL9a* and gma-miR4413b/*GmPPR*, were found in soybean lines, and a miRNA-mediated regulatory network was established [7]. In *B. juncea*, miR167a was found to regulate anther dehiscence by targeting the auxin response factor (*ARF6*/*ARF8*), and miR156a was found to regulate floral transition as well as tapetum development by targeting the *SQUAMOSA promoter binding protein-like* (*SPL*) transcription factors (TFs) [11]. In Ogura-CMS Chinese cabbage (*B. rapa* ssp. *pekinensis*), two novel miRNAs (novel-miR-448 and novel-miR-335), specifically significantly expressed in flower buds, were identified [12]. In addition, it was indicated that the suppressed expression of sucrose transporter *SUC1* and *H^+^-ATPase 6,* targeted by these two novel miRNAs, might cause energy deficiency and pollen abortion [12].

CircRNAs are a novel class of covalently closed and single-stranded endogenous non-coding RNAs (ncRNAs) that are widely expressed in eukaryotes and prokaryotic archaea. Two recent studies demonstrated that circRNAs could act as competing endogenous RNAs (ceRNAs), which are transcripts that communicate with and regulate each other through competing shared miRNA response elements at a post-transcription level [13,14]. An increasing number of ceRNAs have been reported as biomarkers for the diagnosis or therapeutic targets, and circRNA–miRNA–mRNA networks and circRNA-related ceRNA networks have been constructed in normal physiology and diseases [15,16,17]. However, the studies of circRNAs in plants have just begun. Recently, several differentially expressed (DE) circRNAs that are involved in programmed cell death (PCD) and might play an important role in CMS have been found among the soybean CMS line NJCMS1A, and its maintainer NJCMS1B. It indicates that circRNAs may play a role in regulating flower and pollen development, and provide novel insights into the mechanisms of CMS [18]. However, the circRNA–miRNA–mRNA network was not established in the abovementioned study, and the clear interactions of circRNAs, miRNAs, and mRNAs among male reproductive development need further study.

To address these issues and to further understand the mechanisms of CMS, we performed an integrated analysis of circRNAs, miRNAs, and mRNAs expression profiles in *pol* CMS line “Bcpol97-05A” and the fertile line “Bcajh97-01B” in *B. campestris* L. ssp. *chinensis* Makino, syn. *B. rapa* ssp. *chinensis* by whole transcriptome sequencing in this study. We generated the miRNA–mRNA and ceRNA networks by combining the identified and annotated DE RNAs. Our results show that the circRNAs might act as ceRNAs to regulate anther development in *pol* CMS for the first time, which provide novel clues for revealing the molecular mechanisms of male reproductive development and pollen fertility.

## 2. Results

### 2.1. Establishment of the CMS Line in Brassica Campestris

**Figure 1:**
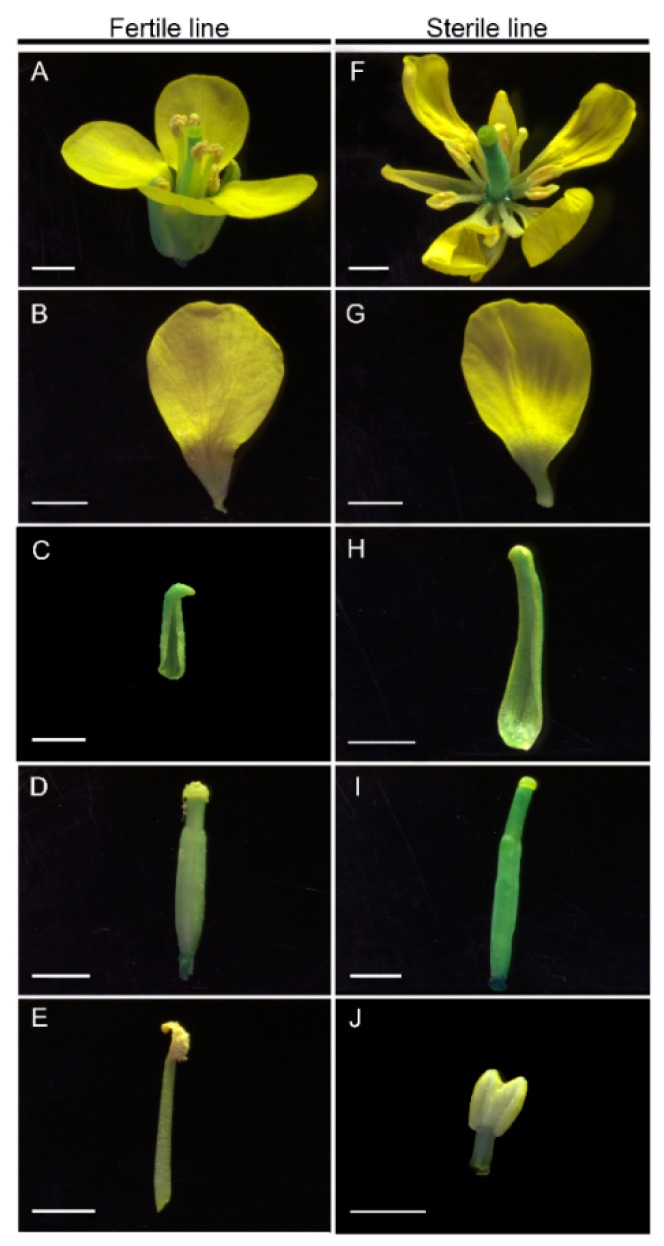
The morphology of flowers of “Bcajh97-01B” (fertile line) and “Bcpol97-05A” (sterile line) of *Brassica campestris*. Scale bars of 2 mm.

**Figure 2:**
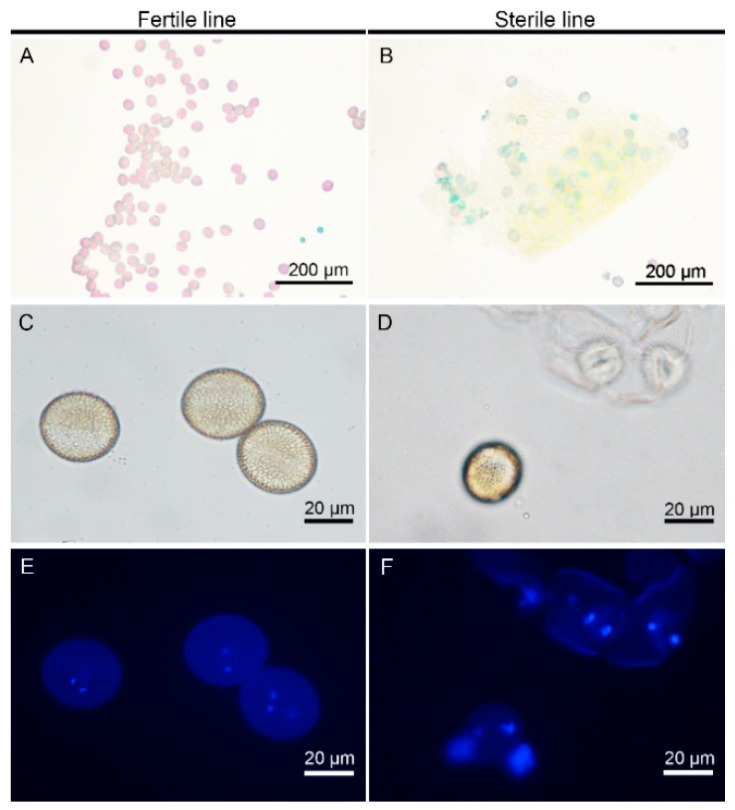
Pollen characteristics of Bcajh97-01B (fertile line) and Bcpol97-05A (sterile line) of *Brassica campestris*. (**A**,**B**) Pollen was dyed with Alexander’s stain. (**C**–**F**) Pollen was dyed with 4’, 6-diamidino-2-phenylindole.

**Figure 3:**
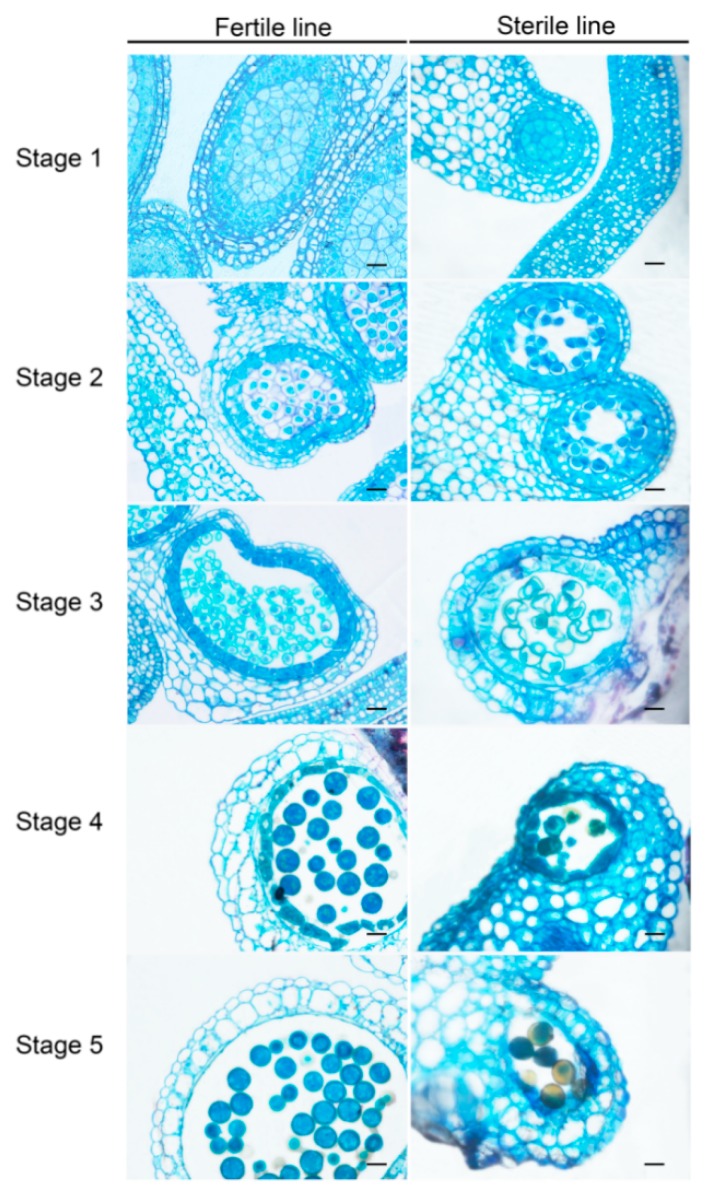
Anther transverse sections of Bcajh97-01B (fertile line) and Bcpol97-05A (sterile line) of *Brassica campestris*. Stage 1, pollen mother cell stage; Stage 2, meiosis stage; Stage 3, uninucleate stage; Stage 4, binucleate stage; Stage 5, mature pollen stage. Scale bars of 20 μm.

To obtain the plant materials with the closest genetic background, we used the fertile plant Bcajh97-01B in the ‘Aijiaohuang’ GMS AB line “Bcajh97-01A/B” as the recurrent parent, and backcrossed Bcajh97-01B to *pol* CMS for eight generations. The height, width, leaf number, leaf color, and other morphology characteristics during the seedling and rosette stage, as well as the sepal, petal, stamen, pistil, and nectary development during the flowering stage of the backcross progenies were observed over successive years. We finally established the male sterile type, *pol* CMS Bcpol97-05A, of which the maintainer line was Bcajh97-01B in *B. campestris*. There was no significant difference in the morphology characteristics, except for in the stamen and male fertility observed between the backcross progenies and the recurrent parent [19]. Compared to the fertile line, the stamens of Bcpol97-05A seemed to have been sapped of their pigment, and took on an albino look. They were also shriveled with no mature pollen grains. In addition, the stamens of Bcpol97-05A seemed shorter and exhibited connected anthers (Figure 1E,J). As there were no obvious mature pollen grains on the surface of the stamens of the sterile line, we crushed the stamens and dyed them with Alexander’s stain in order to observe pollen viability. Compared with the high viability of the pollen in the fertile line, the viability of the pollen from the sterile line was much lower, and about 85% pollen grains were abortive (Figure 2A,B). The morphological characteristics and nuclear development of the pollen grains of the two lines were observed by the 4’, 6-diamidino-2-phenylindole (DAPI) staining using a light microscope and fluorescence microscope, respectively. We found that the pollen of the sterile line displayed an abnormal morphology and had abnormal nuclear development (Figure 2C–F). We also observed the phenotypic characterization of the pollen development in the two lines. As shown in Figure 3, the anther sacs in the sterile line were smaller than those in the fertile line. There was no apparent difference between the pollen development of the two lines until the uninucleate stage. In this stage, the tapetum showed earlier degradation and the microspores displayed vacuolization in the sterile line. Ultimately, much less mature pollen grains were found in the sterile line than in the fertile line in the mature pollen stage (Figure 3).

### 2.2. Identification of circRNAs in the Sterile and Fertile Lines

**Figure 4:**
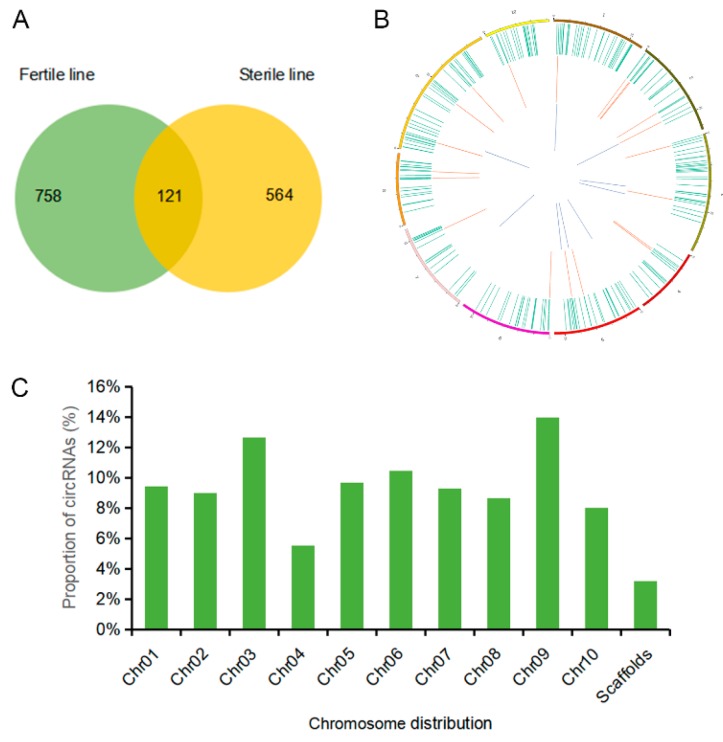
The circRNAs identified in Bcajh97-01B (fertile line) and Bcpol97-05A (sterile line) of *Brassica campestris.* (**A**) The number of circRNAs detected in the sterile line and fertile line. (**B**) Circos plot showing circRNAs on the chromosomes of *B. campestris.* The outmost layer of the ring is the chromosome map of *B. campestris.* The larger inner green ring represents all of the circRNAs detected by RNA-seq. The smaller red ring indicates the differentially expressed circRNAs with up regulation, and the innermost blue layer stands for the down-regulated circRNAs with fold change ≥2 and false discovery rate (FDR) <0.05. (**C**) Histogram of the distribution of circRNAs on the chromosome.

**Figure 5:**
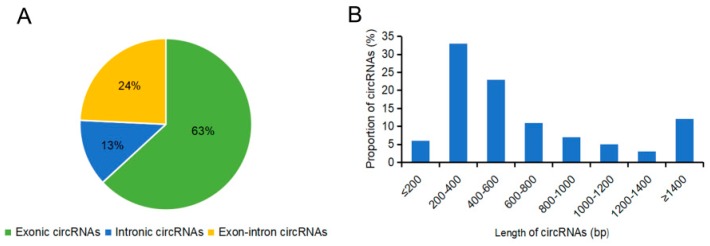
Features of circRNAs identified in Bcajh97-01B (fertile line) and Bcpol97-05A (sterile line) of *Brassica campestris*. (**A**) Types of circRNAs. (**B**) Distribution of the length of circRNAs.

By performing whole genome sequencing, we identified and annotated 1443 circRNAs from the flower buds of the male sterile line and fertile line. In total, 879 circRNAs in the fertile line and 685 circRNAs in the sterile line were discovered to be expressed, and 121 circRNAs were detected in both lines (Figure 4A). The transcripts of the circRNAs were broadly distributed in all of the 10 chromosomes (Figure 4B). Approximately 12.68% of the circRNAs were from chr3, 10.46% from chr6, and 14.00% from chr9, whereas the percentages of circRNAs from other chromosomes were all less than 10% (Figure 4C). The circRNAs were classified into three types, including 911 exonic circRNAs (63.13%), 182 intronic circRNAs (12.61%), and 350 exon-intron circRNAs (24.26%; Figure 5A), and most of them were 200–400 bp in length (Figure 5B).

### 2.3. Identification of DE RNAs in the Sterile Line Compared to the Fertile Line

We analyzed the expression profile data of circRNAs, miRNAs, and mRNAs in order to investigate the possible biological functions of the DE circRNAs and miRNAs between the sterile line and fertile line. The selection criteria for significantly DE circRNAs and mRNAs were a fold change ≥2 and a false discovery rate (FDR) of <0.05. The criteria of the miRNAs were log2(FC) ≥1 and FDR ≤0.05. We identified 31 DE circRNAs (9 up-regulated and 22 down-regulated circRNAs) and 47 DE miRNAs (6 up-regulated and 41 down-regulated miRNAs) in the sterile line. We summarized the top 10 DE circRNAs and miRNAs based on log2FC in Table 1 and Table 2, respectively. In addition, we detected 4779 DE mRNAs (1021 up-regulated and 3758 down-regulated mRNAs) in the sterile line compared to the fertile line.

**Figure 6:**
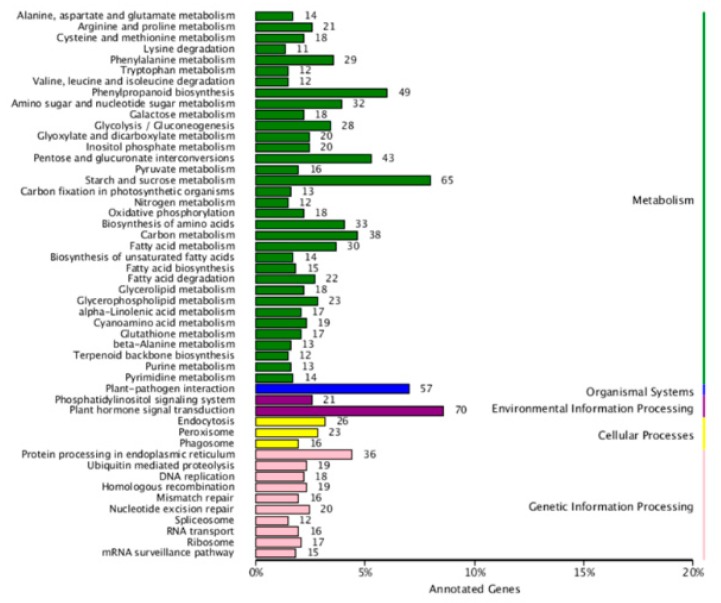
KEGG pathway enrichment analysis of the differentially expressed mRNAs identified in Bcpol97-05A (sterile line) compared to Bcajh97-01B (fertile line) of *Brassica campestris*.

**Figure 7:**
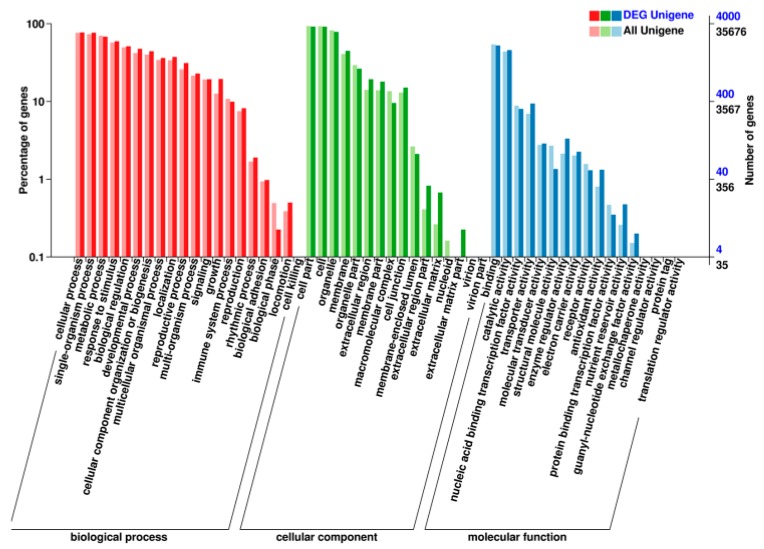
Gene ontology (GO) classification of the differentially expressed mRNAs in the context of DE genes and all of the genes identified in Bcpol97-05A (sterile line) compared to Bcajh97-01B (fertile line) of *Brassica campestris*.

The KEGG pathway enrichment analysis of the DE mRNAs (including the up-regulated and down-regulated mRNAs in the sterile line compared to the fertile line) showed that “starch and sucrose metabolism”, “phenylpropanoid biosynthesis”, and “pentose and glucuronate interconversions” were the most enriched metabolic pathways. The study also showed that most of the DE mRNAs were annotated to be involved in plant hormone signal transduction (Figure 6). We also performed the gene ontology (GO) analysis of the DE mRNAs, and analyzed the enrichment of the secondary functions in the context of DE genes and all genes, reflecting the status of each secondary function in both contexts (Figure 7). In addition, we analyzed the enrichment of each GO term and summarized the most significantly enriched GO terms in categories of the “biological process” (BP) category, “cell component” (CC) category, and “molecular function” (MF). In the BP category, the most significant three nodes were the plant-type cell wall modification, pollen tube growth, and actin filament-based movement. Most of the DE genes were involved in pollen wall development, including pollen exine and inxine formation (Table 3, Table 4 and Table 5; Figures S1–S3).

**Figure 8:**
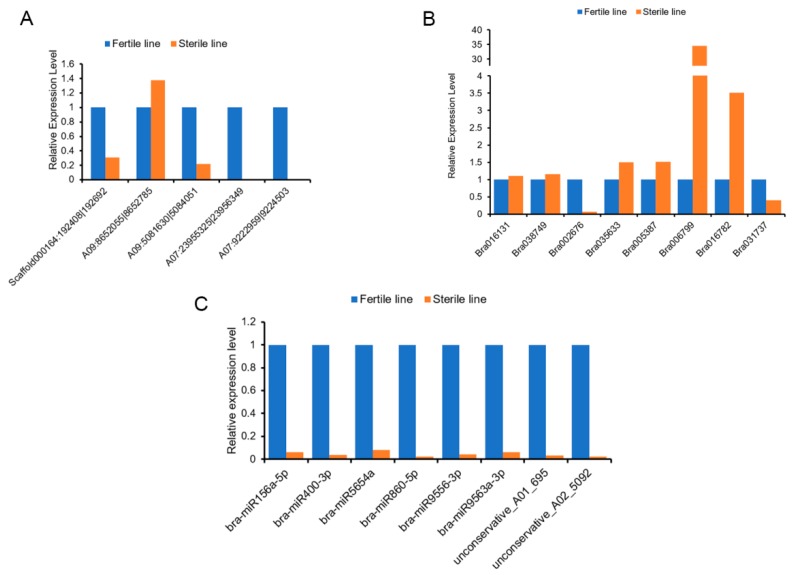
Quantitative real-time PCR validation for the RNA-seq data. (**A**) qRT-PCR validation of circRNAs. (**B**) qRT-PCR validation of mRNAs. (**C**) qRT-PCR validation of miRNAs.

We confirmed the results of RNA-seq by quantitative real-time PCR (qRT-PCR) using the same samples of the sterile and fertile lines. Five circRNAs, eight miRNAs, and eight mRNAs were selected randomly. The qRT-PCR results were consistent with the sequencing results, which demonstrated a high reliability of the RNA profiles (Figure 8).

### 2.4. Construction of the DEmiRNA–DEmRNA Network

**Figure 9:**
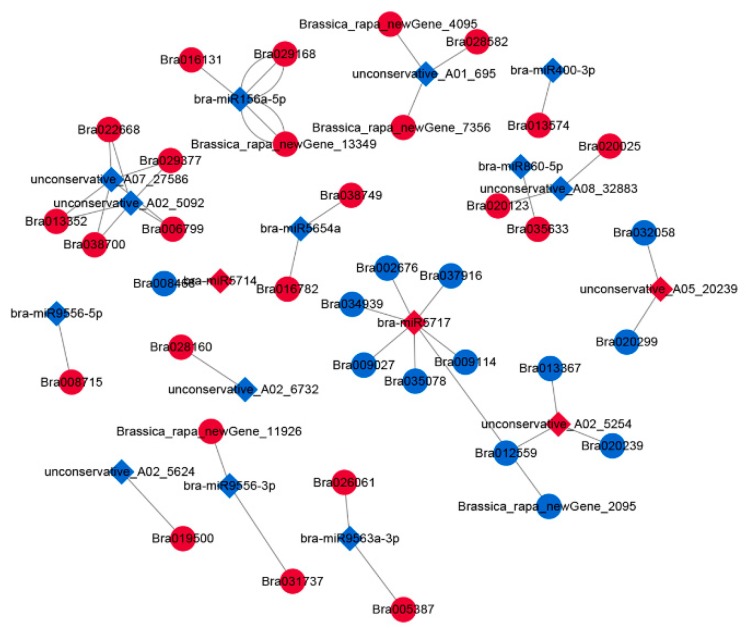
The view of DEmiRNA–DEmRNA network involved in anther development in Bcajh97-01B (fertile line) and Bcpol97-05A (sterile line) of *Brassica campestris*. The network includes 18 miRNAs and 37 mRNAs. miRNA and mRNA are indicated as diamonds and ellipses, respectively. The red color represents the up-regulated expression and the blue color represents the down-regulated expression.

**Figure 10:**
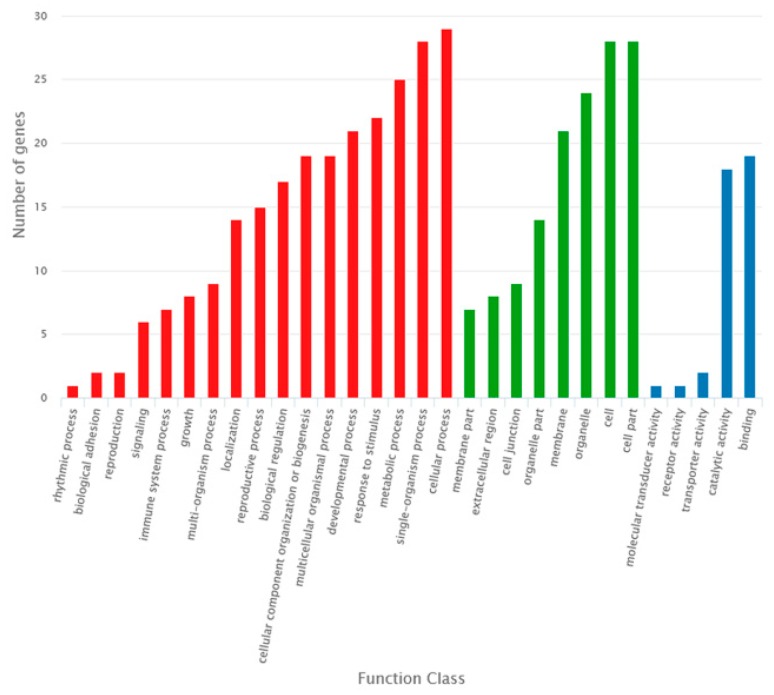
Gene ontology classification of potential targets of miRNAs in the DEmiRNA–DEmRNA network in Bcajh97-01B (fertile line) and Bcpol97-05A (sterile line) of *Brassica campestris.*

To better understand the gene regulatory network during the anther development of *pol* CMS plants, we identified putative DEmiRNA–DEmRNA interactions using TargetFinder software. In total, we obtained 170 DEmiRNA–DEmRNA interactions. We selected the DEmiRNA–DEmRNA pairs of 18 miRNAs and 37 mRNAs, in which the miRNAs and the corresponding mRNAs had the opposite expression and constructed the miRNA-mediated regulatory network using Cytoscape (Figure 9). To investigate the potential functions of the miRNAs, we performed a GO analysis of the putative target mRNAs in this network (Figure 10). The genes were enriched for a total of 30 terms. The top three enriched GO-BP terms were “cellular process”, “single-organism process”, and “metabolic process”. In the cellular component category, the top three terms were “cell part”, “cell”, and “organelle”. Additionally, “catalytic activity” and “binding” were the most highly presentative terms in the molecular function category.

In our constructed network, miRNAs primarily function as regulators of the genes involved in pathways like sugar metabolism (bra-miR9563a-3p, unconservative_A02_5254, bra-miR9556-3p, bra-miR156a-5p, bra-miR9556-5p, unconservative_A08_32883, and bra-miR5717), PCD (unconservative_A02_5254, unconservative_A05_20239, bra-miR9556-3p, and bra-miR9556-5p), lipid metabolism (bra-miR9563a-3p, unconservative_A08_32883, bra-miR5717, unconservative_A05_20239, unconservative_A08_32883, and bra-miR9556-3p), and pollen tube growth (bra-miR9563a-3p and bra-miR5717; Table 6). And almost all of the transcripts were phytohormone-related and could be involved in plant hormone metabolism and/or signaling pathways, suggesting that most of miRNAs that we identified were phytohormone-related (Table 7). For example, bra-miR9556-5p might be involved in the salicylic acid mediated signaling pathway and brassinosteroid biosynthetic process by targeting Bra008715 (Table 7).

### 2.5. Construction of the DEcircRNA–DEmiRNA–DEmRNA Network

We obtained two DEcircRNA–DEmiRNA interactions that consisted of one up-regulated circRNA (A02:23507399|23531438) and two down-regulated putative miRNA (unconservative_A02_5092 and unconservative_A07_27586) targets in the sterile line compared to the fertile line by TargetFinder software. Increasing evidence has shown that some specific circRNAs can act as miRNAs sponges, which act to isolate and prevent miRNAs from binding to the target genes. To investigate the regulation of ceRNA regulation in anther development and to identify anther development related circRNAs, we constructed the DEcircRNA–DEmiRNA–DEmRNA network based on the miRNAs with both circRNAs and mRNAs.

**Figure 11:**
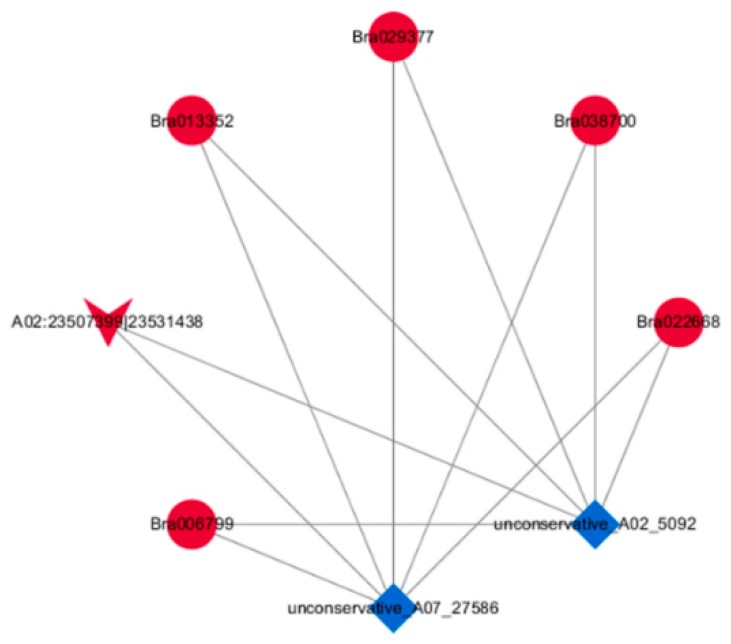
View of the DEcircRNA–DEmiRNA–DEmRNA triple network involved in anther development in Bcajh97-01B (fertile line) and Bcpol97-05A (sterile line) of *Brassica campestris.* The network includes one circRNA, two miRNAs, five mRNAs, and 11 edges. circRNA, miRNA, and mRNA are indicated as a deformed V, diamonds, and ellipses, respectively. The red color represents the up-regulated expression, and the blue color represents the down-regulated expression.

The ceRNA network consisted of one circRNA, two miRNAs, five mRNAs (Bra006799, Bra013352, Bra022668, Bra029377, and Bra038700), and 10 interaction pairs (Figure 11). In the constructed ceRNA network, two novel miRNAs, unconservative_A02_5092 and unconservative_A07_27586, were correlated negatively (Spearman correlation ≤−0.5) with the corresponding targets, including circRNAs and mRNAs. In the sterile line, each of the five mRNAs was down-regulated, possibly via targeted decay or cleavage by miRNAs. The inhibition of the two miRNAs might be caused by the up-regulation of A02:23507399|23531438, an exon-intron circRNA of 24,039 bp long, that was generated from the intergenic region of the host gene. A02:23507399|23531438 might bind to unconservative_A02_5092 and unconservative_A07_27586 via sequence complementation, and could affect the binding of miRNA to the five target mRNAs. We summarized the targeted gene ID, *Arabidopsis* homologue ID, and the roles of homologues in the ceRNA network in Table 8 [20,21,22,23,24,25,26].

## 3. Discussion

### 3.1. Whole-Transcriptome Sequencing Enriches Understanding of Mechanisms of Anther Development and Male Sterility

Male sterility, like CMS, has become a crucial breeding tool for producing new crop hybrids. It provides important materials to study the mechanisms of anther development, and supports an abundant food supply in the world.

With the development of high-throughput sequencing methods, abundant DE genes related to anther development have been identified in the sterile and fertile lines by deep sequencing in *pol* CMS plants, which deepen our understanding of anther development and male sterility [5,6]. In *pol* CMS in *B. napus*, some dramatically down-regulated unigenes controlling anther development were identified in the sterile buds, and seven down-regulated callose synthase genes were detected [5]. In our study, about 79% of DE genes were down-regulated in the sterile line. We found a callose related gene, Bra005387, which could be involved in the biological process of defense response by callose deposition. Abnormal callose deposition during meiosis could lead to the degeneration of pollen mother cells at the early meiosis stage, and could result in the complete collapse of pollen grains [27]. Furthermore, transcriptomic data demonstrated that many DE genes in *pol* CMS in *B. napus* were related to hormonal signal transduction pathways, and the genes related to pentose–glucuronate interconversions and starch–sucrose metabolism were significantly enriched [6]. In our study, we also found that “starch and sucrose metabolism” and “pentose and glucuronate interconversions” were the most enriched metabolic pathways. However, the two studies mentioned above only explored the mechanisms of *pol* CMS system at the mRNA level, but the mechanisms underlying *pol* CMS on the whole-transcriptome have yet to be reported. Increasing new evidence shows the importance of ncRNAs, including miRNAs, long non-coding RNAs, small interfering RNAs, and circRNAs, functioning in the male reproductive development in flowering plants [18,28,29,30]. Among these ncRNAs, only the function of miRNAs regulating gene expression at a post-transcriptional level in CMS biogenesis has been investigated in detail [31].

Here, we studied the alterations under *pol* CMS biogenesis in *B. campestris* at a whole-transcriptome level, and we identified several anther development related RNAs, including mRNAs, circRNAs, and miRNAs. We also re-constructed the putative regulatory network involving these RNA molecules, which will deepen and enrich our understanding of anther development and male fertility control.

### 3.2. miRNAs Contribute to Pollen Wall Development

In plants, miRNAs could play an important role in regulating male reproductive development. An increasing number of miRNAs related to male reproductive development have been found by comparing the microtranscriptome data of CMS and fertile lines in multiple plant species. The identified miRNAs mainly acted as regulators of TF encoding genes [11,12,32]; PCD [18]; phytohormone-related pathways [8,9]; and a series of metabolism processes, like lipid metabolism [8], amino acid metabolism [18], sulphur metabolism [11,32], and energy metabolism [12]. Among the identified miRNAs, miR156/7a existed frequently by targeting *SQUAMOSA promoter binding protein-like* (*SPL*). In our study, the identified miRNAs were also mainly involved in processes like phytohormone-related pathways, PCD, and lipid metabolism. However, no miRNAs that regulate the expression of TF genes like *SPL* and auxin response factor, besides miR156a-5p, were found. We also did not find miRNAs to be involved in regulating amino acid metabolism, sulphur metabolism, and energy metabolism. Most of the miRNAs identified in our study were involved in sugar metabolism, lipid metabolism, PCD, and so on, influencing the formation of the functional pollen wall.

The pollen grain is surrounded by a sculpted multiple-layer wall, namely the pollen wall, which consists of two independent parts, the outer wall (exine) and the inner wall (intine). The appropriate genetic regulation of PCD in tapetum is essential to the production of functional pollen with a normal exine pattern [33,34]. In our study, we noticed that the tapetum developed aberrantly by cytologically observing the anthers in different stages of the two lines. In addition, we found six miRNAs that could function as regulators of PCD of tapetum, including bra-miR9556-5p, unconservative_A02_5254, bra-miR9556-3p, unconservative_A02_5092, unconservative_A07_27586, and unconservative_A05_20239. They affect the development of tapetum cells during anther development. The abnormal expression of the targeted genes, like Bra032058, Bra008715, and Bra031737, in the sterile line might prevent tapetum from the normal PCD process, providing precursors for tryphine and sporopollenin, leading to abortive pollen formation.

In this study, we also identified large amounts of miRNAs functioning in sugar metabolism, for example, galactose metabolism, L-arabinose metabolism, xyloglucan metabolism, and pectin metabolism, which could regulate the development of intine composed of a variety of hydrolases, hydrophobic proteins, cellulose, hemicellulose, and pectic polymers.

MiRNA156, first reported in *Arabidopsis*, is one of the most highly conserved miRNA families in plants, and it plays a key role in floral development and male fertility [35,36,37]. The SPL genes targeted by miR156/7 as well as the non-targeted *SPL8* are necessary in order to maintain the male fertility and are required for the sporogenous cell formation of anthers in *Arabidopsis.* The target genes of miR156 identified in our study were Bra016131 and Brassica_rapa_newGene_13349 involved in cell wall biosynthesis, indicating that miR156 has other targets in *B. campestris.* The homologue to *Arabidopsis* of Bra016131 is AT1G71690, a glucuronoxylan 4-O-methyltransferase-like protein involved in the xylan biosynthetic process and plant-type secondary cell wall biogenesis. The up-regulation of Bra016131 and Brassica_rapa_newGene_13349 caused by miR156a-5p might influence the xylan biosynthetic process and glucuronoxylan metabolic process, producing pollen grains with an abnormal pollen wall and function. These results demonstrated the important roles of miR156 in anther development.

In addition, miR9563, which has been found only in Brassica including *B. campestris* and *B. napus* [38,39], has also been detected as being differentially expressed between the fertile and sterile line in our study. MiR9563 has been identified as the regulator of fatty acid metabolism, including fatty acid synthesis and fatty acid β-oxidation. In *B. napus,* bra-9563a-p3 and bra-MIR9563b-p5_1ss3GA were found to regulate ADSL1 (stearoyl-CoA desaturase/delta-9 desaturase) and ACO (acyl-CoA oxidase) expression, respectively [39]. We found that bra-miR9563a-3p could regulate pollen wall development and pollen tube development by targeting the Bra005387 involved in sugar metabolism, including monosaccharide (galactose and L-arabinose) and polysaccharide (xyloglucan, rhamnogalacturonan I, and rhamnogalacturonan II) metabolisms. Besides being involved in sugar metabolism and the biological process of the defense response by callose deposition, Bra005387 might also be capable of regulating pollen tube development. These results demonstrate that miRNAs could play key roles in pollen wall development and affect the functional pollen formation by regulating sugar metabolism. Our miRNA–mRNA network unravels some novel functions of miRNAs, expands the list of fertility related miRNA, and provides new clues for exploring the mechanisms of anther development.

### 3.3. CeRNA Networks Could Provide New Sights into the Regulatory Roles of ncRNAs during Anther Development

Increasing evidence has demonstrated that circRNAs could function as efficient miRNA sponges to offset the repression of mRNA mediated by miRNA [40]. Recently, the regulatory axes of circRNAs, miRNAs, and mRNAs were demonstrated in various diseases [15,41]. However, the circRNA–miRNA–miRNA triple regulatory network has not been widely constructed in plants. To explore the ceRNA network and the functions of circRNAs in anther development in *B. campestris*, we constructed the triple circRNA–miRNA–mRNA network. From the network, we could see that A02:23507399|23531438 might be a special male-sterility circRNA and act as a miRNA sponge by targeting miRNA unconservative_A06_21945 and unconservative_Scaffold000096_42992 in order to regulate the expression of Bra002750, which could be involved in the cuticular wax biosynthetic process and pollen sperm cell differentiation. Additionally, Bra002750 could also be involved in the metabolic process of very long-chain fatty acids, which are precursors of sporopollenin and pollen coat (also named tryphine in Brassicaceae). The homologue of Bra002750 to *Arabidopsis* is AT5G57800 (*FLP1*/*WAX2*/*CER3*), which is a transmembrane protein with similarities to the sterol desaturase family at the N-terminus, and to the short-chain dehydrogenase/reductase family at the C-terminus.

The surface of the pollen grains of the loss-of-function mutant of *FLP1* was smoother than that of the wild-type *Arabidopsis*, because the excess tryphine filled in the cavities of exine and covered the pollen grains [22]. The *flp1* mutant was observed to be defective in sporopollenin, and to have aberrant bacula and tactum [22]. Additionally, the transgenic cucumbers of *CsWAX2*, the *AtWAX2* homologue, showed a significantly decreased fertility [42]. The transmission electron microscopy (TEM) observation indicated that lipid droplets in the tryphine of *CsWAX2ox-6* (over-expression lines) pollen were more numerous compared with the wild-type pollen, while those of *CsWAX2i-2* (RNAi lines) pollen were rarely existent.

In our study, the up-regulated A02:23507399|23531438 suppressed the expression of unconservative_A06_21945 and unconservative_Scaffold000096_42992, leading to the up-regulation of Bra002750. The up-regulated Bra002750 might affect the synthesis of tryphine and sporopollenin in the exine of the sterile line, producing abortive pollen.

Our study demonstrated that ceRNA networks existed in *pol* CMS in *B. campestris,* and A02:23507399|23531438 could function in anther development by acting as the “miRNA sponge” of unconservative_A06_21945 and unconservative_Scaffold000096_42992, thereby regulating the expression of Bra002750 and the biosynthesis of tryphine and sporopollenin.

## 4. Materials and Methods

### 4.1. Plant Materials and Sample Collection

The sterile line, Bcpol97-05A, and its maintainer line, Bajh97-01B, in *B. campestris* ssp. *chinensis* cv. Aijiaohuang, were used in this study. They were both cultivated in the same experimental plot in Zhejiang University (Hangzhou, Zhejiang, China). After flowering, the fertile and sterile plants were identified, and the inflorescences (with all of the different developmental stages) were sampled (three biological replicates) from the individual plants for each material, snap-frozen in liquid nitrogen, and kept at −75 °C for further use.

### 4.2. Morphological and Cytological Observation

The flower organs were observed by a stereomicroscope. Alexander dying and DAPI staining were performed as previously described [43]. The sample processing procedures of the semi-thin section observation, which were the same as those in the TEM observation, were performed as described in Lin‘s study [44]. The pollen grains were dyed with Alexander’s stain and DAPI, and the semi-thin sections were observed by a light microscope.

### 4.3. Total RNA Extraction and Detection

The total RNA of the inflorescences was isolated from individual plants according to the instructions of the Trizol kit (Life Technologies, Gaithersburg, MD, USA), and for each material, the RNAs used for the whole-transcriptome analysis were mixed from 30 individual plants. Each line had three biological repetitions. The integrity and purity of the total RNA were analyzed. The detection methods included the following: (1) Nanodrop detection—detection of the purity of RNA samples (OD260/280 ≥1.8; OD260/230 ≥0.5). (2) Qubit 2.0 detection—accurate quantification of the concentration of the RNA samples (total RNA concentration ≥65 ng/µL). (3) Agilent 2100 bioanalyzer test—the integrity of the RNA sample is checked to ensure that the qualified sample is used for sequencing (the RIN value of total RNA is ≥7.0, 28S/18S ≥1.0; the baseline of the spectrum is not uplifted; the 5S peak is normal).

### 4.4. RNA Library Construction and Sequencing

After verifying the RNA sample’s quality, according to the following steps, the libraries of circRNAs, miRNAs, and mRNAs were constructed, respectively, as follows: the rRNA was removed using the epicentre Ribo-Zero™ kit. The liner RNAs were digested using RNase R (this step was skipped in the construction of mRNA library and miRNA library). The treated RNAs were interrupted randomly by adding a fragmentation buffer (a fragment reagent). Using the fragmented RNA as a template, the first strand was synthesized using random hexamers, followed by buffer, dNTPs, RNase H, and DNA polymerase I, to synthesize the second strand of cDNA, which was purified by AMPure XP beads. The sticky ends of the DNA were repaired to the blunt ends by T4 DNA polymerase and Klenow DNA polymerase. The A tail was added to the 3’ end, and the sequencing linker was ligated. Afterwards, the fragments were selected according to size with AMPure XP beads, followed by the degradation of the cDNA second strand containing U using USER. Finally, the cDNA library was obtained by a polymerase chain reaction. After the construction of the library, the concentration and insert size of the library were detected using Qubit2.0 and Agilent 2100, respectively. The effective concentration of the library was accurately quantified by the Q-PCR method so as to ensure the library quality (determined to be >2 nM). After detection, the different libraries were pooled based on the target machine data volume, and were sequenced on the Illumina Hi-Seq platform of BioMarker Technologies (Beijing, China).

### 4.5. Sequencing Quality Control and Biological Analysis

To ensure the accuracy of the information analysis, the quality control of the original data was performed in order to obtain a high-quality sequence (i.e., clean reads). The standard of the original sequence quality control is as follows: (1) removing the joint-containing reads, (2) filtering to remove low-quality data, and (3) removing the reads containing an N (undetermined base information) ratio that is greater than 5%. The clean data were aligned to the *Brassica rapa* genome in order to obtain the mapped data. We tested the quality of the library based on the mapped data, including the insert lengths and randomness. We performed the prediction of the RNAs, circRNA-binding sites, miRNA-binding sites, expression analysis of different RNA samples, DE gene expression analysis, KEGG analysis, and GO analysis.

### 4.6. Identifcation of RNAs

We predicted the circRNAs using the find_circ software. First, the find_circ software took 20 bp as the anchor point at both ends of the reads on the genomic alignment, then it compared the anchor points as independent reads to the *Brassica rapa* genome, and found the only matching site where the alignment positions of the two anchors were reversed in the linear direction. Afterwards, the reads of the anchor would be extended until the junction position of the circular RNA was found [45]. If the sequences on both sides were GT/AG splicing signals, respectively, they would be determined as circular RNA. We identified the known miRNAs by aligning the sequences of the mapped reads with the sequences of the mature miRNAs in miRBase (v21). If their sequences were totally identical, then the reads were identified as known miRNAs. We predicted the novel miRNAs using miRDeep2 software [46]. Based on the *Brassica rapa* genome sequence, we identified the new transcripts and genes by splicing the mapped reads with Cufflinks software, and comparing this with the original genomic annotation information.

### 4.7. Identification of DE RNAs

We used DESeq software to detect the DE RNAs. The screening criteria of DE circRNAs and mRNA were a fold change of ≥2 and FDR ≤0.05. The criteria of miRNAs were log2(FC) ≥1 and FDR ≤0.05. To avoid the false positive problem, we used the well-known Benjamini–Hochberg correction method to correct the *p*-value of the original hypothesis test, and finally the FDR was used as the key indicator for the differential expression of RNA screening.

### 4.8. Target Gene Prediction, GO Analysis, and KEGG Analysis of the Target Genes

We identified the targets of miRNAs using TargetFinder, based on the known miRNAs, the newly predicted miRNAs, and the gene sequence information in *Brassica rapa*. As circRNAs contain multiple miRNA binding sites, the miRNA target gene prediction methods can be used to identify the circRNAs that bind to miRNAs, and the functions of the circRNAs can be elucidated based on the functional annotation of the miRNA target genes. The predicted target gene sequence was aligned with the GO database and KEGG database using BLAST software, so as to obtain the annotation information of the target genes, respectively [47,48].

### 4.9. qRT-PCR Analysis of RNAs

The levels of DE circRNAs, miRNAs, and mRNAs in the fertile and sterile lines were detected by qRT-PCR in a CFX96 Real-Time System (Bio-Rad, Hercules, CA, USA). The total RNA was extracted from the inflorescences of the fertile line and sterile line using RNAiso Plus (TaKaRa, Dalian, China). The cDNA used for the qRT-PCR analysis of mRNA, and circRNA was synthesized using a PrimeScript^TM^ RT reagent Kit with a gDNA Eraser (TaKaRa, Dalian, China). The cDNA used for the qRT-PCR analysis of miRNA was synthesised by a Mir-X^TM^ miRNA First-Strand Synthesis Kit (TaKaRa, Dalian, China). The primer sequences of the selected circRNAs, miRNAs, and mRNAs are listed in Supplementary Tables S1–S3, and the reverse primer of the selected miRNAs was the primer provided in Mir-X^TM^ miRNA First-Strand Synthesis Kit (TaKaRa, Dalian, China). We conducted qRT-PCR using TaKaRa TB Green™ Premix Ex Taq™ II (TaKaRa, Dalian, China). The expression levels of circRNAs, miRNAs, and circRNAs were defined based on the threshold cycle, and the relative expression levels were calculated using the 2^−ΔΔ*C*t^ method.

### 4.10. Construction of miRNA–mRNA and circRNA–miRNA–mRNA Networks

The miRNA–mRNA and circRNA–miRNA­–mRNA networks were constructed according to the predicted miRNA–mRNA and circRNA–miRNA pairs using Cytoscape software. CircRNA, miRNA, and mRNA are indicated as a deformed V, diamonds, and ellipses, respectively. The red color represents the up-regulated expression, and the blue color represents the down-regulated expression.

## Figures and Tables

**Table 1:** Top 10 differentially expressed circRNAs in Bcpol97-05A (sterile line) compared to Bcajh97-01B (fertile line) of *Brassica campestris.*

circRNA ID	Host Gene ID	Type	FDR	Log2FC	Status
A02:2218194|2218504	–	Intergenic region	0.003556881	−5.326588229	Down
Scaffold000212:46573|47221	–	Intergenic region	0.017264214	−3.898467742	Down
A01:1717686|1718114	Bra011550	Intron	0.024218267	−3.569160763	Down
A08:17623961|17626439	–	Intergenic region	0.027029147	−3.459397447	Down
A02:2598446|2599366	–	Intergenic region	0.03231053	−3.277304232	Down
A03:23207122|23207987	Bra012618	Exon	0.034434505	−3.211114134	Down
A05:21205001|21205592	Bra027353	Exon	0.036783873	−3.141740631	Down
A08:14993249|14994216	Bra010648	Exon	0.039392172	−3.068861972	Down
A03:25432732|25433123	Bra019257	Intron	0.036783873	3.141740631	Up
A03:20154550|20158652	–	Intergenic region	0.007140366	4.712416081	Up

**Table 2:** Top 10 differentially expressed miRNAs in Bcpol97-05A (sterile line) compared to Bcajh97-01B (fertile line) of *Brassica campestris.*

miRNA ID	FDR	Log2FC	Status
unconservative_A07_27582	8.05 × 10^−9^	−5.710103115	Down
unconservative_A06_21945	6.89 × 10^−11^	−5.240482741	Down
unconservative_Scaffold000096_42992	6.89 × 10^−11^	−5.240482741	Down
unconservative_Scaffold000777_48008	6.89 × 10^−11^	−5.240482741	Down
unconservative_A02_5624	7.51 × 10^−11^	−4.410730302	Down
unconservative_A10_42699	1.57 × 10^−7^	−4.101491939	Down
unconservative_A06_21764	3.57 × 10^−21^	−4.07323988	Down
unconservative_A06_23706	3.61 × 10^−8^	−4.053517585	Down
unconservative_A08_32883	3.23 × 10^−18^	−3.88622319	Down
unconservative_A03_12748	4.87 × 10^−16^	−3.37012631	Down

**Table 3:** Top 10 GO terms in BP category of differentially expressed genes in Bcpol97-05A (sterile line) compared to Bcajh97-01B (fertile line) of *Brassica campestris*.

GO Term ID	GO Term Description	Unigene Numeber	DE Gene Number	Expected Number	KS
GO:0009827	plant-type cell wall modification	1070	360	121.45	<1 × 10^−30^
GO:0009860	pollen tube growth	1366	407	155.05	<1 × 10^−30^
GO:0030048	actin filament-based movement	271	85	30.76	2.60 × 10^−10^
GO:0010584	pollen exine formation	326	103	37	9.30 × 10^−10^
GO:0045490	pectin catabolic process	193	54	21.91	6.80 × 10^−7^
GO:0048235	pollen sperm cell differentiation	116	40	13.17	2.40 × 10^−6^
GO:0015770	sucrose transport	66	25	7.49	1.70 × 10^−5^
GO:0009821	alkaloid biosynthetic process	42	13	4.77	1.80 × 10^−5^
GO:1901700	response to oxygen-containing compound	11730	1436	1331.44	1.90 × 10^−5^
GO:0009737	response to abscisic acid	4007	511	454.82	3.60 × 10^−5^

The “Unigene number” line, “DE Gene Number” line, “Expected Number” line, and “KS” line display the number of unigenes, number of differentially expressed genes, expected number annotated to the related corresponding GO term, and the *p*-value of the KS test, respectively.

**Table 4:** Top 10 GO terms in molecular function (MF) category of differentially expressed genes in Bcpol97-05A (sterile line) compared to Bcajh97-01B (fertile line) of *Brassica campestris*.

GO Term ID	GO Term Description	Unigene Numeber	DE Gene Number	Expected Number	KS
GO:0003785	actin monomer binding	22	13	2.52	1.20 × 10^−5^
GO:0030599	pectinesterase activity	182	51	20.87	1.20 × 10^−5^
GO:0045330	aspartyl esterase activity	102	33	11.7	1.60 × 10^−5^
GO:0090353	polygalacturonase inhibitor activity	14	5	1.61	3.20 × 10^−5^
GO:0008705	methionine synthase activity	12	5	1.38	5.40 × 10^−5^
GO:0019863	IgE binding	65	17	7.45	6.90 × 10^−5^
GO:0009044	xylan 1,4-beta-xylosidase activity	31	15	3.55	0.0001
GO:0004350	glutamate-5-semialdehyde dehydrogenase activity	11	7	1.26	0.00014
GO:0004601	peroxidase activity	225	52	25.8	0.00023
GO:0004349	glutamate 5-kinase activity	13	7	1.49	0.00023

The “Unigene number” line, “DE Gene Number” line, “Expected Number” line, and “KS” line display the number of unigenes, number of differentially expressed genes, expected number annotated to the related corresponding GO term, and the *p*-value of the KS test, respectively.

**Table 5:** Top 10 GO terms in the CC category of differentially expressed genes in Bcpol97-05A (sterile line) compared to Bcajh97-01B (fertile line) of *Brassica campestris*.

GO Term ID	GO Term Description	Unigene Numeber	DE Gene Number	Expected Number	KS
GO:0003785	actin monomer binding	22	13	2.52	1.20 × 10^−5^
GO:0030599	pectinesterase activity	182	51	20.87	1.20 × 10^−5^
GO:0045330	aspartyl esterase activity	102	33	11.7	1.60 × 10^−5^
GO:0090353	polygalacturonase inhibitor activity	14	5	1.61	3.20 × 10^−5^
GO:0008705	methionine synthase activity	12	5	1.38	5.40 × 10^−5^
GO:0019863	IgE binding	65	17	7.45	6.90 × 10^−5^
GO:0009044	xylan 1,4-beta-xylosidase activity	31	15	3.55	0.0001
GO:0004350	glutamate-5-semialdehyde dehydrogenase activity	11	7	1.26	0.00014
GO:0004601	peroxidase activity	225	52	25.8	0.00023
GO:0004349	glutamate 5-kinase activity	13	7	1.49	0.00023

The “Unigene number” line, “DE Gene Number” line, “Expected Number” line, and “KS” line display the number of unigenes, number of differentially expressed genes, expected number annotated to the related corresponding GO term, and the *p*-value of the KS test, respectively.

**Table 6:** Differentially expressed miRNAs and their target genes in the DEmiRNA–DEmRNA network involved in anther development in Bcajh97-01B (fertile line) and Bcpol97-05A (sterile line) of *Brassica campestris*.

miRNA ID	Status	Gene ID	Protein	Biological Process
bra-miR9563a-3p	Down	Bra005387	UDP-glucose 4-epimerase, UDP-arabinose 4-epimerase	Sugar metabolism; pollen tube development; defense response by callose deposition
unconservative_A02_5254	Up	Bra012559	Xyloglucan:xyloglucosyl transferase	Callose metabolic process
		Bra013367		
		Bra020239	Unknown	Cellulose biosynthetic process; cell redox homeostasis regulation; tapetum programmed cell death
bra-miR9556-3p	Down	Brassica_rapa_newGene_11926	Unknown	Flavonoid biosynthesis process
		Bra031737	Unknown	Tapetum programmed cell death
unconservative_A05_20239	Up	Bra020299	Unknown	Glucuronoxylan metabolic process; xylan biosynthetic process
		Bra032058	Unknown	Tapetum programmed cell death
bra-miR156a-5p	Down	Brassica_rapa_newGene_13349	Unknown	Vegetative to reproductive phase transition of meristem; glucuronoxylan metabolism; xylan biosynthetic process
		Bra016131	Unknown	Unknown
bra-miR9556-5p	Down	Bra008715	Unknown	Lignin metabolic process; glucuronoxylan biosynthetic process; cell wall organization process; tapetum programmed cell death
unconservative_A08_32883	Down	Bra020025	Unknown	Lipid metabolic process; xylan metabolic process; cell wall biogenesis
		Bra020123	Unknown	Flavonoid biosynthetic process
bra-miR5717	Up	Bra002676	Glycerophosphodiester phosphodiesterase	Pollen tube growth process; pollen wall development; starch metabolism; lipid metabolism
		Bra009027	Unknown	Pollen tube growth process; pollen wall development
		Bra035078	Unknown	Pollen tube growth process; pollen wall development
		Bra034939	N-acylphosphatidylet-hanolamine-specific phospholipase D	Pollen wall development
		Bra037916	S-adenosylmethionine-dependent methyltransferase	Pollen wall development; pectin metabolic process

**Table 7:** miRNAs involved in the phytohormone-related biological process in Bcajh97-01B (fertile line) and Bcpol97-05A (sterile line) of *Brassica campestris.*

miRNA ID	Target Gene ID	Phytohormone-Related Biological Process
unconservative_A05_20239	Bra032058	Be involved in salicylic acid biosynthetic process and response to salicylic acid
bra-miR9563a-3p	Bra005387	Be involved in abscisic acid-activated signaling pathway and response to ethylene
unconservative_A02_5254	Bra013367	Response to auxin
bra-miR5717	Bra034939	Response to abscisic acid
	Bra037916	Response to abscisic acid
bra-miR9556-5p	Bra008715	Be involved in salicylic acid mediated signaling pathway and brassinosteroid biosynthetic process

**Table 8:** Differentially expressed mRNA putative targets of ‘A02:23507399|23531438′ in Bcajh97-01B (fertile line) and Bcpol97-05A (sterile line) of *Brassica campestris.*

Gene ID	Homologue to *Arabidopsis*	Description of the Homologue to *Arabidopsis*	Role of the Homologue to *Arabidopsis*	Reference
Bra006799	AT5G57800	Encodes a transmembrane protein with similarity to the sterol desaturase family at the N-terminus and to the short-chain dehydrogenase/reductase family at the C-terminus.	Be involved in cuticle membrane and wax biosynthesis, influencing pollen fertility as well as plant biotic and abiotic stress responses, etc.	[20,21,22]
Bra013352	AT4G18810	NAD(P)-binding Rossmann-fold superfamily protein	Unknown	–
Bra022668	AT5G53730	Late embryogenesis abundant (LEA) hydroxyproline-rich glycoprotein family	Unknown	–
Bra029377	AT5G23580	A Member of a unique family of enzymes containing a single polypeptide chain with a kinase domain at the amino terminus and a putative calcium-binding EF hands structure at the carboxyl terminus	Be involved in plant response to salt stress, a Ca (2+) -dependent protein kinase balancer in abscisic acid signaling	[23,24,25]
Bra038700	AT3G12145	A novel leucine-rich repeat protein	Interacts directly with MADS domain transcription factor during *Arabidopsis thaliana* flower development	[26]

## Supplementary Materials

Supplementary Materials can be found at https://au-mynotebook.labarchives.com/share/My%2520Notebook/MjcuM3w2ODQwOS8yMS9UcmVlTm9kZS80MTMzMzE4NzI2fDY5LjM=. Figure S1. Enriched directed acyclic gragh of top GO terms in BP category of differentially expressed genes in ‘Bcpol97-05A’ (sterile line) compared to ‘Bcajh97-01B’ (fertile line) of Brassica campestris. Figure S2. Enriched directed acyclic gragh of top GO terms in CC category of differentially expressed genes in ‘Bcpol97-05A’ (sterile line) compared to ‘Bcajh97-01B’ (fertile line) of Brassica campestris. Figure S3. Enriched directed acyclic gragh of top GO terms in MF category of differentially expressed genes in ‘Bcpol97-05A’ (sterile line) compared to ‘Bcajh97-01B’ (fertile line) of Brassica campestris.

## Abbreviations

circRNAs	circular RNAs
ncRNAs	non-coding RNAs
miRNA	microRNA
ceRNAs	competing endogenous RNAs
CMS	cytoplasmic male sterility
DE	differentially expressed
GO	gene ontology
PCD	programmed cell death
GMS	genic male sterility
*pol*	Polima
TFs	transcription factors
DAPI	4’,6-diamidino-2-phenylindole
FDR	false discovery rate
BP	biological process
CC	cell component
MF	molecular function
qRT-PCR	quantitative Real-Time PCR
TEM	transmission electron microscopy
