# Supplementary material for: CircRNA Expression Pattern and ceRNA and miRNA–mRNA Networks Involved in Anther Development in the CMS Line of Brassica campestris
Source: Int J Mol Sci. 2019 Sep 27;20(19):4808. doi: 10.3390/ijms20194808 (PMC6801457; doi:10.3390/ijms20194808)
Supplement: Supplementary file 1 [file ijms-20-04808-s001.zip › Supplementary Files/Supplementary_tables.docx]

**Table S1.** Primers used in the qRT-PCR analysis of randomly selected differently expressed circRNAs.

| **Gene** | **Primer Sequences (5’-3’)** |
| --- | --- |
| *Scaffold000164:192408\|192692-F* | CACCATGAAAGGGTCCAGTCC |
| *Scaffold000164:192408\|192692-R* | GACAGACACAGAAGCGCACC |
| *A09:8652055\|8652785-F* | TGCCAGCGGTATGAATCGAC |
| *A09:8652055\|8652785-R* | ATAGCGCAGACGAGTTCGAG |
| *A09:5081630\|5084051-F* | CTGAGACCAAAGGAGTGCCA |
| *A09:5081630\|5084051-R* | TAGTGCTTTGTGCATGGCTG |
| *A07:23955325\|23956349-F* | CGCTGCAGAAACGGATTGTT |
| *A07:23955325\|23956349-R* | GGTCTCAAAGACGGAAGCGA |
| *A07:9222959\|9224503-F* | TGTGCATGTGGTTGGACCAG |
| *A07:9222959\|9224503-R* | CCAGCTCACAACAGCTACCG |

**Table S2.** Primers used in the qRT-PCR analysis of randomly selected differently expressed miRNAs.

| **Gene** | **Primer Sequences (5’-3’)** |
| --- | --- |
| *bra-miR156a-5p-F* | TGACAGAAGAGAGTGAGCAC |
| *bra-miR400-3p-F* | GACTTATAATGATCTCATGAA |
| *bra-miR5654a-F* | ATAAATCCCAAGCATCATCCA |
| *bra-miR5714-F* | AGACTCTACGACATCAAGAAAC |
| *bra-miR860-5p-F* | ATGTAGTCCAATCTATTGAAG |
| *bra-miR9556-3p-F* | TCTACTTTCACCAATTGGCCT |
| *bra-miR9563a-3p-F* | TAAAAGTTAAGAGACAAGTTA |
| *unconservative_A01_695-F* | CGGCTCTGATACCAATTGATG |
| *unconservative_A02_5092-F* | TTGACAGAAGATAGAGAGC |
| *unconservative_A02_5254-F* | GTTCCCTTTAACGCTTCATTG |

**Table 3.** Primers used in the qRT-PCR analysis of randomly selected differently expressed mRNAs.

| ***Gene*** | **Primer Sequences (5’-3’)** |
| --- | --- |
| *Bra016131-F* | ACAACTAACCTCCACTTTCCG |
| *Bra016131-R* | AGATCTGGATAGTTTTGGCCG |
| *Bra038749-F* | TGTCCTTTTCTAGCCGTTACC |
| *Bra038749-R* | CCAACATTCACATATGCCACG |
| *Bra002676-F* | ACAATGGAGCAAGTGGAGAC |
| *Bra002676-R* | GCAGAATCATGGCAGAAAGC |
| *Bra035633-F* | GCTGCAATACCATCAACGAG |
| *Bra035633-R* | CTTTGTCTTATGCCACACGC |
| *Bra031737-F* | GACGAGATGACAGTGGGAAC |
| *Bra031737-R* | TGGTCTGTGGAGTTTATGGTG |
| *Bra005387-F* | AATGTTGGTACCGGGAAAGG |
| *Bra005387-R* | TTTATATCAACCCCTGTCGCC |
| *Bra006799-F* | AGGACTCATCGTATTGCTTGTG |
| *Bra006799-R* | TGGGATTTGGAACAGGAGATG |
| *Bra012559-F* | GACATTGCTCTTGACGGTTG |
| *Bra012559-R* | ATGGTTGAGTAATCGCTGGTC |
| *Bra029168-F* | CAGAATTCCCAAATCGAAACCG |
| *Bra029168-R* | TTCCCTTTCCCTTTCTTAGCC |
| *Bra016782-F* | AAAGCGAGGGTCTACAAAGG |
| *Bra016782-R* | CGTACAAAGACCACAAAGACAC |
