# Supplementary figures and images for: CircRNA Expression Pattern and ceRNA and miRNA–mRNA Networks Involved in Anther Development in the CMS Line of Brassica campestris
Source: Int J Mol Sci. 2019 Sep 27;20(19):4808. doi: 10.3390/ijms20194808 (PMC6801457; doi:10.3390/ijms20194808)

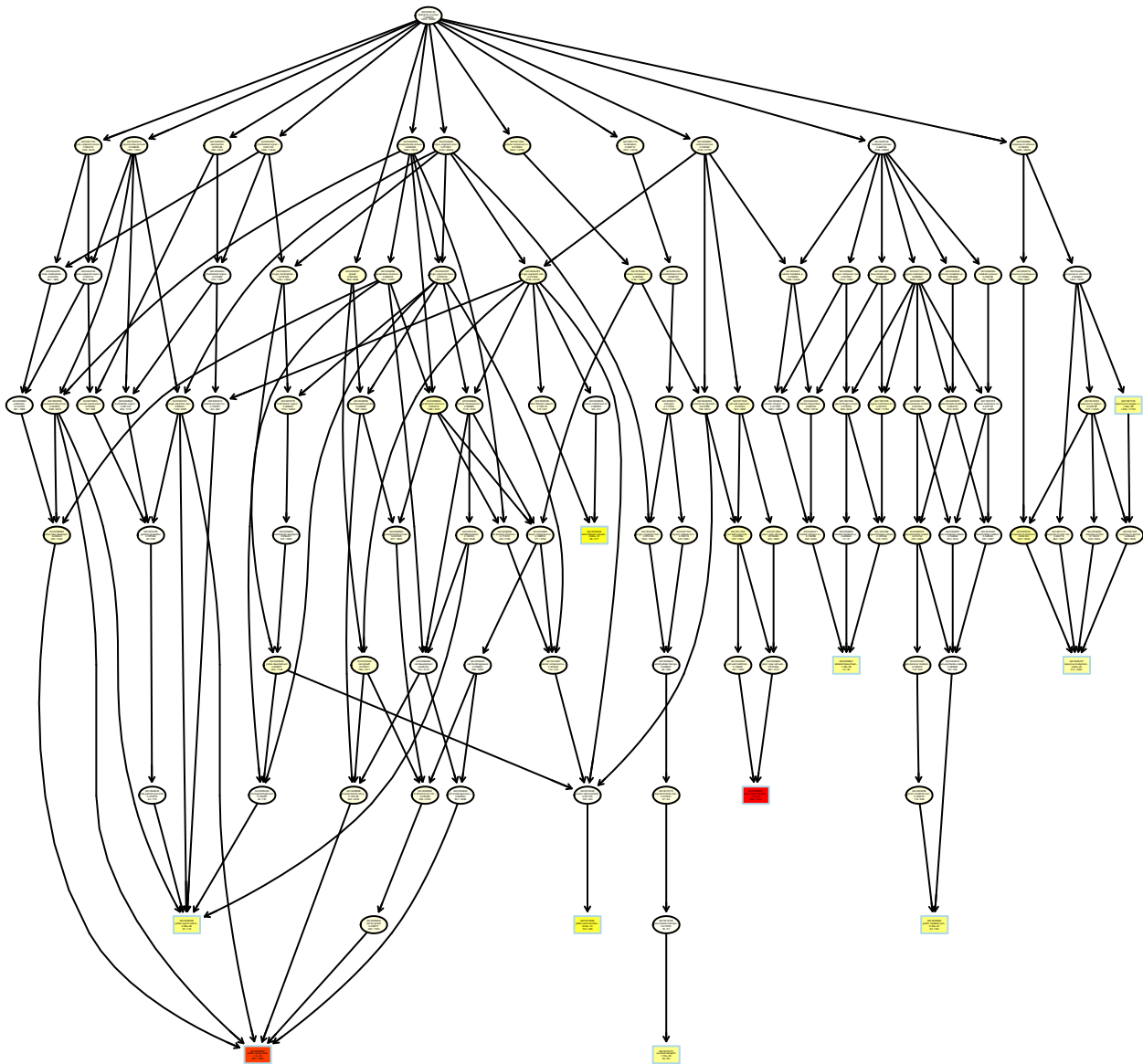

Supplement: Supplementary file 1 [file ijms-20-04808-s001.zip › Supplementary Files/Supplementary_Figure_1..pdf]

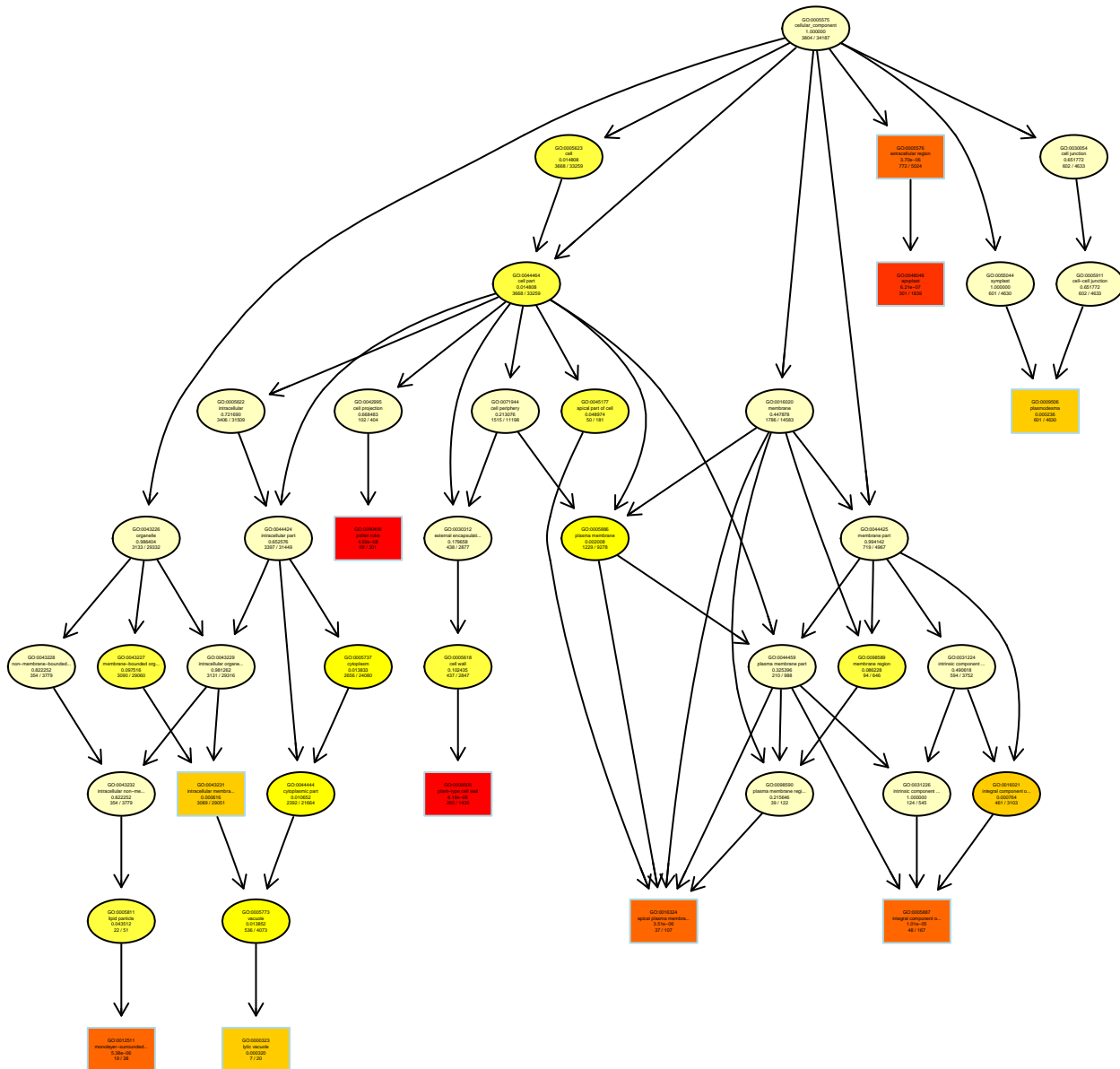

Supplement: Supplementary file 1 [file ijms-20-04808-s001.zip › Supplementary Files/Supplementary_Figure_2..pdf]

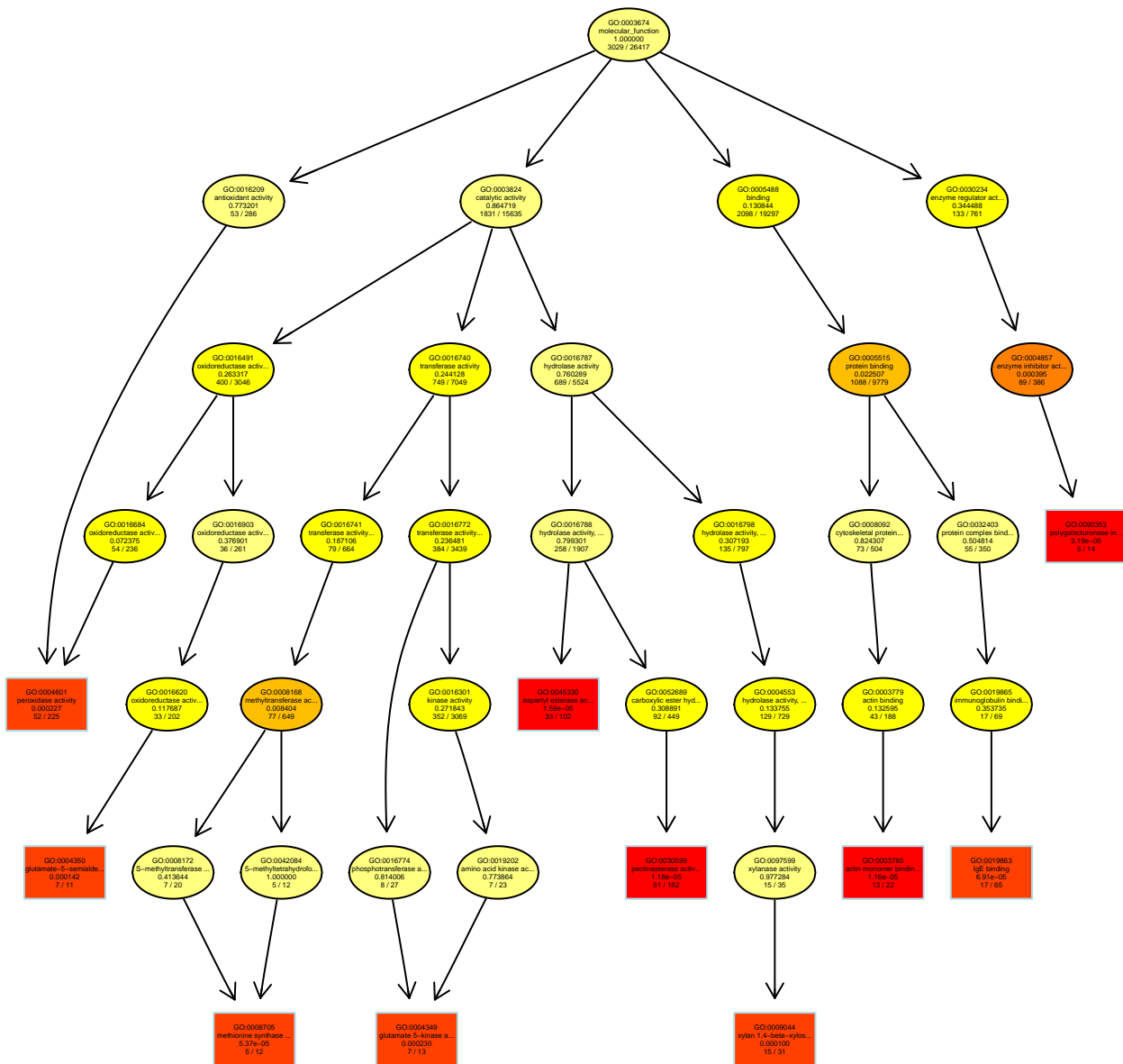

Supplement: Supplementary file 1 [file ijms-20-04808-s001.zip › Supplementary Files/Supplementary_Figure_3..pdf]
